# Supplementary material for: Ethanol abolishes vigilance-dependent astroglia network activation in mice by inhibiting norepinephrine release
Source: Nat Commun. 2020 Dec 2;11:6157. doi: 10.1038/s41467-020-19475-5 (PMC7710743; doi:10.1038/s41467-020-19475-5)
Supplement: Supplementary file 1 — Supplementary Information [file 41467_2020_19475_MOESM1_ESM.pdf]

Supplementary Information

Ethanol abolishes vigilance-dependent astroglia network activation in mice by inhibiting norepinephrine release

Liang Ye[1,7], Murat Orynbayev[1,7], Xiangyu Zhu[1,2], Eunice Y. Lim[1,3], Ram R. Dereddi[4], Amit Agarwal[4,5], Dwight E. Bergles[5,6], Manzoor A. Bhat[1,3] and Martin Paukert[1,3,*]

1, Department of Cellular and Integrative Physiology, University of Texas Health Science Center at San Antonio, San Antonio, TX, United States of America

2, Xiangya School of Medicine, Central South University, Changsha, Hunan, China

3, Center for Biomedical Neuroscience, University of Texas Health Science Center at San Antonio, San Antonio, TX, United States of America

4, The Chica and Heinz Schaller Research Group, Institute for Anatomy and Cell Biology, Heidelberg University, Heidelberg, Germany

5, Solomon H. Snyder Department of Neuroscience, Johns Hopkins University School of Medicine, Baltimore, MD, United States of America

6, Johns Hopkins Kavli Neuroscience Discovery Institute

7, These authors contributed equally

*, paukertm@uthscsa.edu

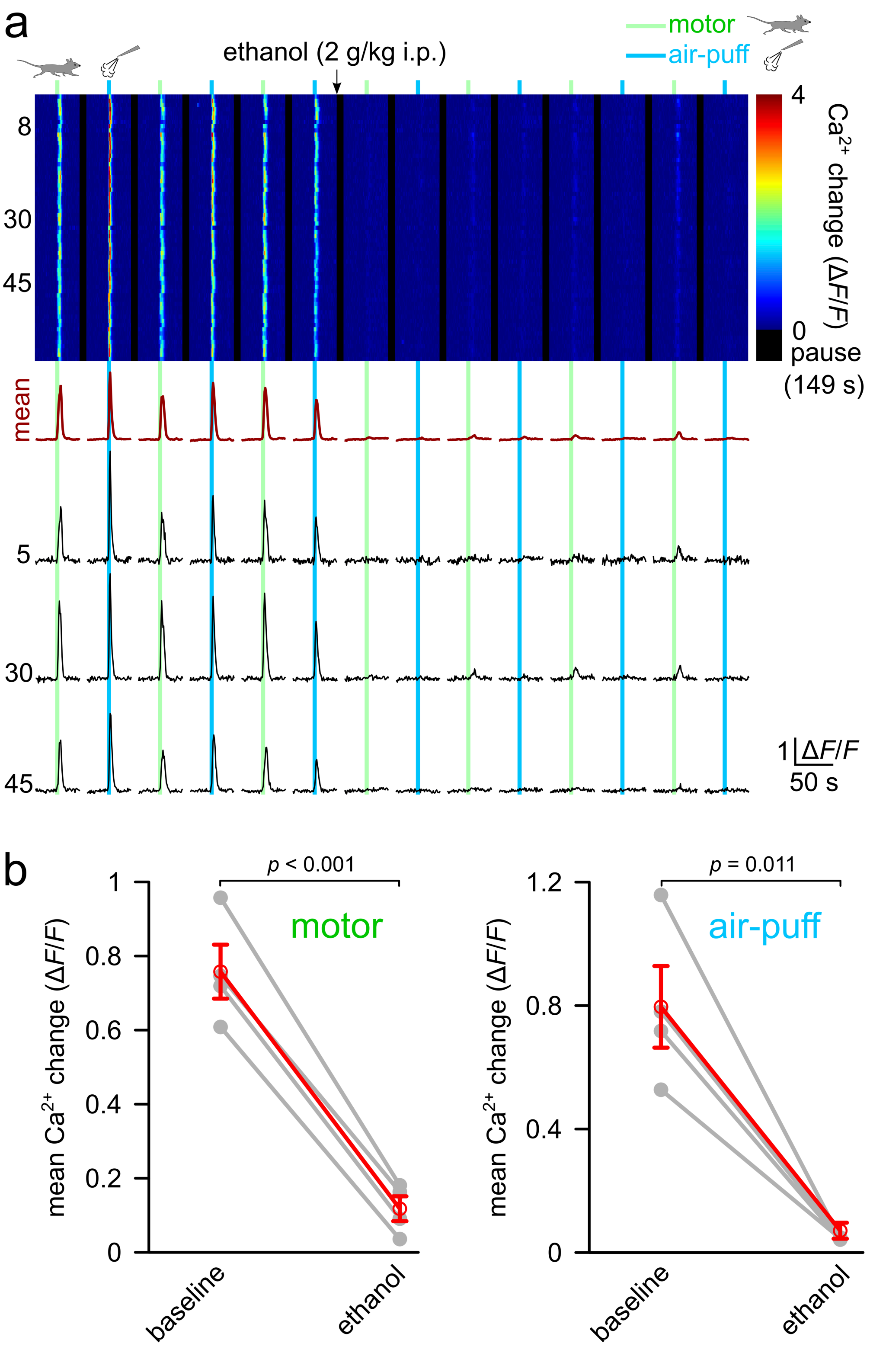

Supplementary Fig. 1 Ethanol inhibits vigilance-dependent BG Ca^{2+} elevations also in cerebellar vermis, whether induced by locomotion or by air-puff. a upper, Pseudocoloured plot represents Ca^{2+} responses of all ROI-defined BG process clusters around consecutive enforced locomotion trials (green bars) or air-puffs (blue bars). Arrow highlights the time point of i.p. injection of 2 mg/kg ethanol. **lower**, Average Ca^{2+} response of all processes (dark red) and traces of Ca^{2+}

dynamics from three representative BG process clusters. **b left**, Population data showing the effect of ethanol on mean changes of locomotion-induced Ca^{2+} elevation (mean of first three locomotion trials compared to mean of last three locomotion trials). Red symbols represent mean ± SEM ($\Delta F/F$). n = 4 mice, paired, two-tailed Student's t-test ($t(3) = 13.607$). **right**, Population data showing the effect of ethanol on mean changes of air-puff-induced Ca^{2+} elevation (mean of first three air-puff trials compared to mean of last three air-puff trials). Red symbols represent mean ± SEM ($\Delta F/F$). n = 4 mice, paired, two-tailed Student's t-test ($t(3) = 5.691$). Source data are provided as a Source Data file.

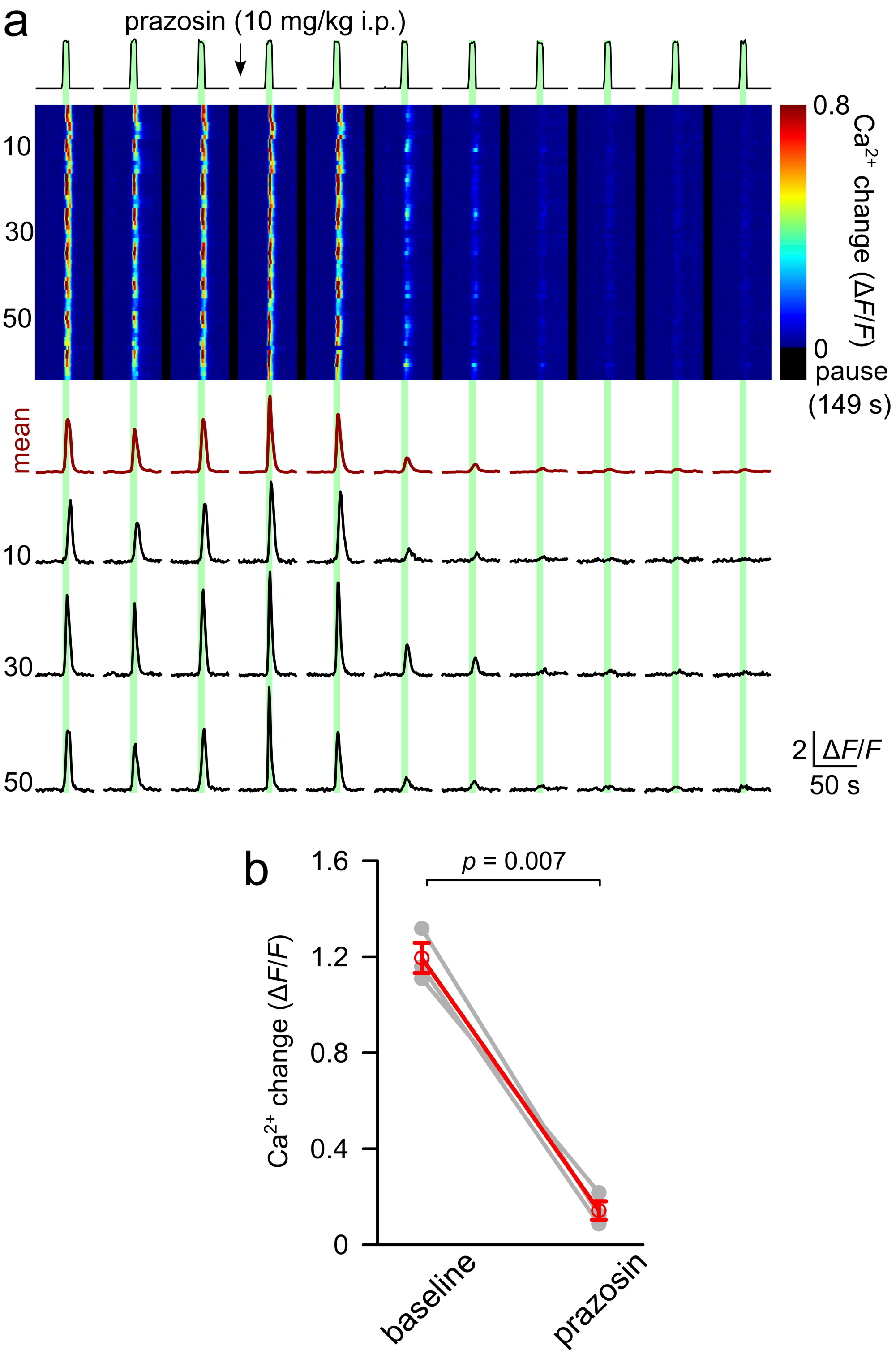

Supplementary Fig. 2 Vigilance-dependent BG Ca^{2+} elevations are inhibited by the α_1-adrenergic receptor antagonist prazosin. a upper, Pseudocoloured plot represents Ca^{2+} responses of all ROI-defined BG process clusters around consecutive enforced locomotion trials (green bars). Arrow highlights the time point of i.p. injection of 10 mg/kg prazosin. **lower**, Average Ca^{2+} response of all processes (dark red) and traces of Ca^{2+} dynamics from three representative BG process clusters. **b** Population data showing the effect of prazosin on mean changes of locomotion-induced Ca^{2+}

elevation (mean of first three trials compared to mean of last two trials). Red symbols represent mean ± SEM ($\Delta F/F$). n = 3 mice, paired, two-tailed Student's t-test ($t(2) = 11.982$). Source data are provided as a Source Data file.

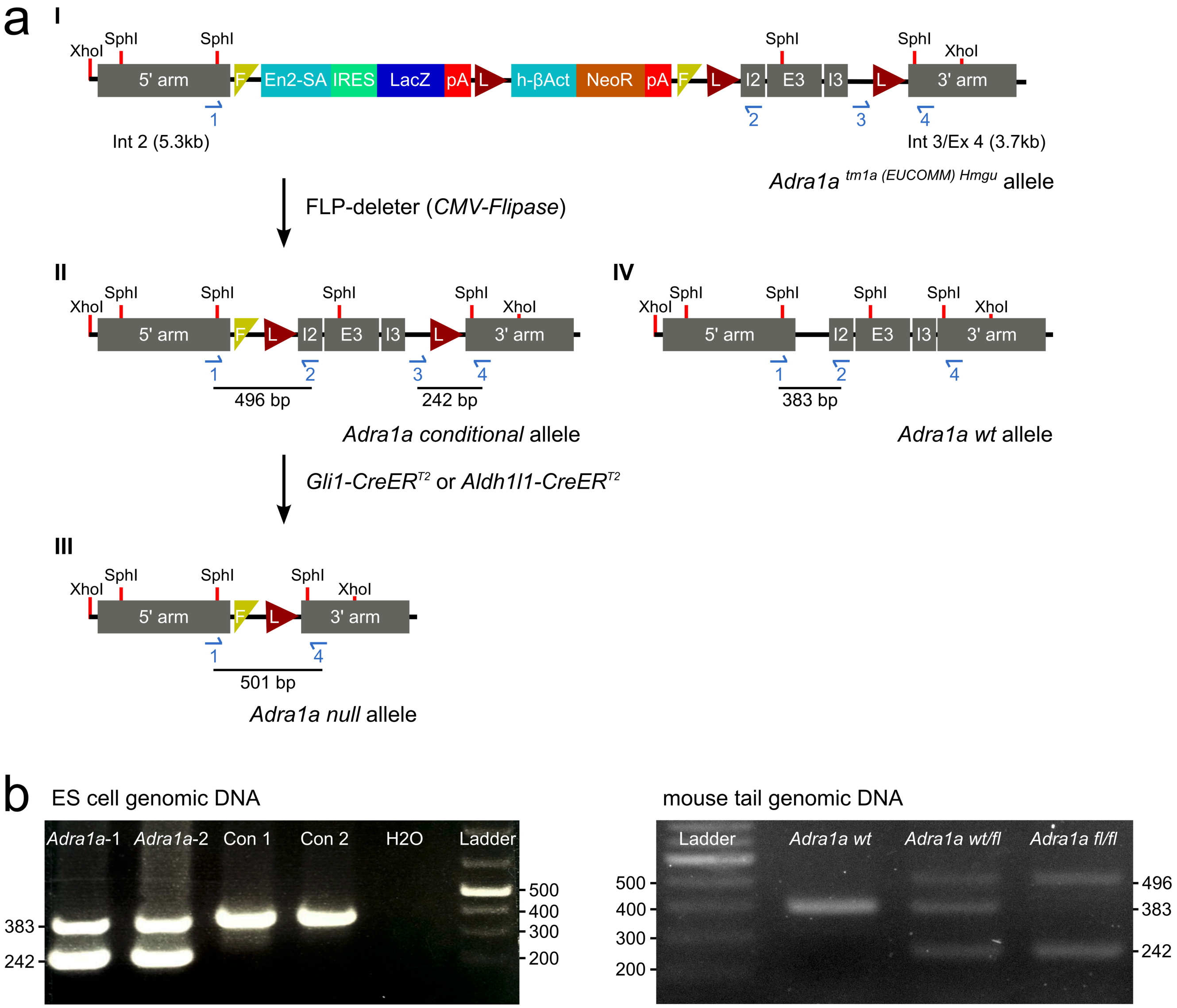

Supplementary Fig. 3 Generation of *Adra1a*^{cKO} mouse line. a The structure of knock-out first LacZ reporter based conditional Adra1a allele in *Adra1a* $^{tm1\ (EUCOMM)\ Hmgu}$ mice. (I) The Adra1a knock-out first allele after homologous recombination in murine ES cells harbors 5.3kb and 3.7kb 5' (5' arm, part of intron 2) and 3' (3' arm, part of intron 3 and exon 4) homology arms from Adra1a locus, the engrailed2 splice acceptor (En2-SA), AN Internal ribosome entry site (IRES) driven b-galactosidase reporter gene cassette (IRES-LacZ-pA) and human b-actin promoter driven neomycin resistance cassette (h-bAct-NeoR-pA). Both LacZ reporter and neomycin resistance cassettes were flanked by two FRT sites (shown as yellow triangles with F). A part of intron 2 (I2), the entire exon 3 (E3) and a part of intron 3 (I3) were flanked by loxP sites (shown as brown triangles with L) to generate Adra1a floxed allele. (II) Flipase mediated *in vivo* site-specific recombination to remove LacZ reporter and neomycin resistance cassettes and generate Adra1a conditional allele. (III) Adra1a null allele after astrocyte specific Cre recombinases mediated *in vivo* recombination. (IV) Genomic structure of Adra1a wildtype allele. Blue arrows (numbered 1-4) indicate locations of various primers used to confirm proper targeting of Adra1a locus or genotyping Adra1a mutant mice by PCR based strategies. **b left**, PCR with primers

#1-4 on genomic DNA extracted from 2 targeted (*Adra1a*-1 and *Adra1a*-2: 242 and 383 bp) and 2 wild type control (Con1 and Con2: 383 bp) ES cell clones confirms correct targeting of Adra1a locus. **right**, A PCR with primers #1-4 on genomic tail DNA from wild type ($Adra1a^{wt/wt}$: 383 bp), heterozygous ($Adra1a^{wt/cKO}$: 242, 383 and 496 bps) and homozygous ($Adra1a^{cKO/cKO}$: 242 and 496 bps) mice, further confirms correct targeting of Adra1a locus. These results have been reproduced in more than 200 genotyping testings.

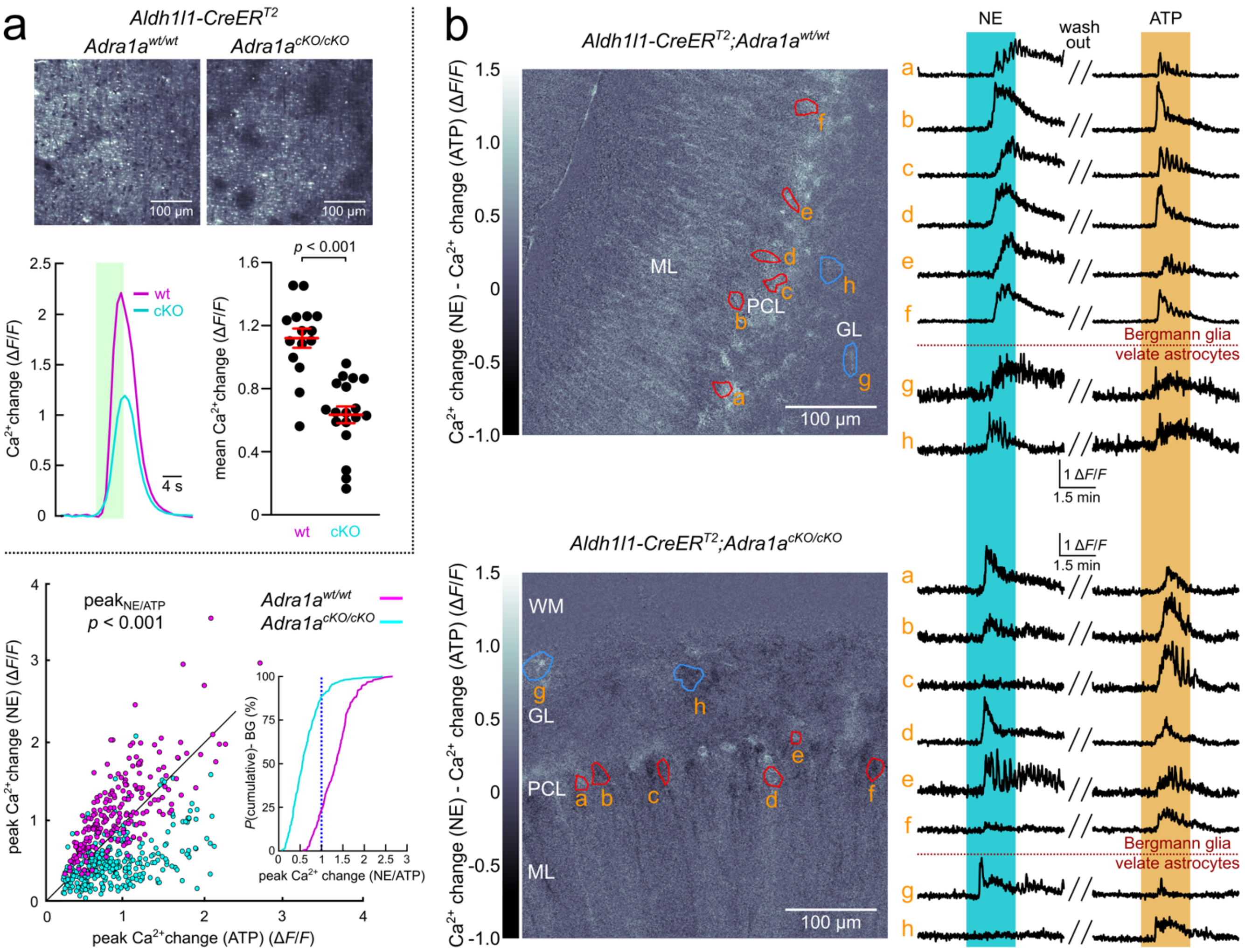

Supplementary Fig. 4 Ca^{2+} responsiveness of cerebellar BG and velate astrocytes to NE is conferred by α$_{1A}$-adrenergic receptor. a upper, Representative BG locomotion-induced Ca^{2+} response plots of *Aldh1l1-CreER^{T2};Ai95;Adra1a$^{wt/wt}$* and *Aldh1l1-CreER^{T2};Ai95;Adra1a$^{cKO/cKO}$* mice. **lower left**, Overlay of averaged Ca^{2+} change traces of wt mice (15 FOVs from 5 mice) and cKO mice (18 FOVs from 6 mice). **lower right**, Population data of mean Ca^{2+} changes. Red symbols indicate mean ± SEM. Unpaired, two-tailed Student's *t*-test ($t(31) = 6.015$). **b** Responsiveness of BG to bath application of NE and ATP in acute cerebellar slices determined by 2P Ca^{2+} imaging of *Aldh1l1-CreER^{T2};Ai95;Adra1a$^{wt/wt}$* or *Aldh1l1-CreER^{T2};Ai95;Adra1a$^{cKO/cKO}$* mice (PND 120 - 150). **upper left**, Representative visualization (see methods) of the difference between responses to NE and ATP of *Adra1a$^{wt/wt}$* BG and velate astrocytes (9 independent experimental repetitions were obtained with similar results). The brighter structures appear the stronger the response to NE compared to ATP, and vice versa. Red circumferences indicate representative ROIs enclosing individual BG somata in the Purkinje cell layer. Blue circumferences indicate representative ROIs enclosing individual velate astrocyte somata in the granular layer. **upper right**, Corresponding Ca^{2+} change traces determined for each ROI depicted on the left. Blue bar highlights episode of NE application, orange bar highlights episode of ATP application. Double-line blanks 11 additional minutes of agonist washout with aCSF. **lower middle and right**, Same presentation as above for *Adra1a$^{cKO/cKO}$* BG and velate astrocytes in

the *Aldh1l1-CreER^{T2};Ai95* background (9 independent experimental repetitions were obtained with similar results). **lower left**, Population data represented as two-dimensional plot comparing peak Ca^{2+} change in individual BG in response to NE or ATP, respectively. Colour of circle fillings highlights genotype as indicated. Diagonal line aids in identifying identical responses to NE and ATP. Kruskal-Wallis test compared the ratio of Ca^{2+} responses of individual BG to NE / ATP among genotypes (wt, 214 BG from 9 slices from 3 mice; cKO, 308 BG from 9 slices from 3 mice). Inset illustrates the cumulative probability distribution of individual BGs' responses to NE normalized to their respective response to ATP. The dashed line corresponds to the equi-response line. GL, granular layer; PCL, Purkinje cell layer; ML, molecular layer. Source data are provided as a Source Data file.

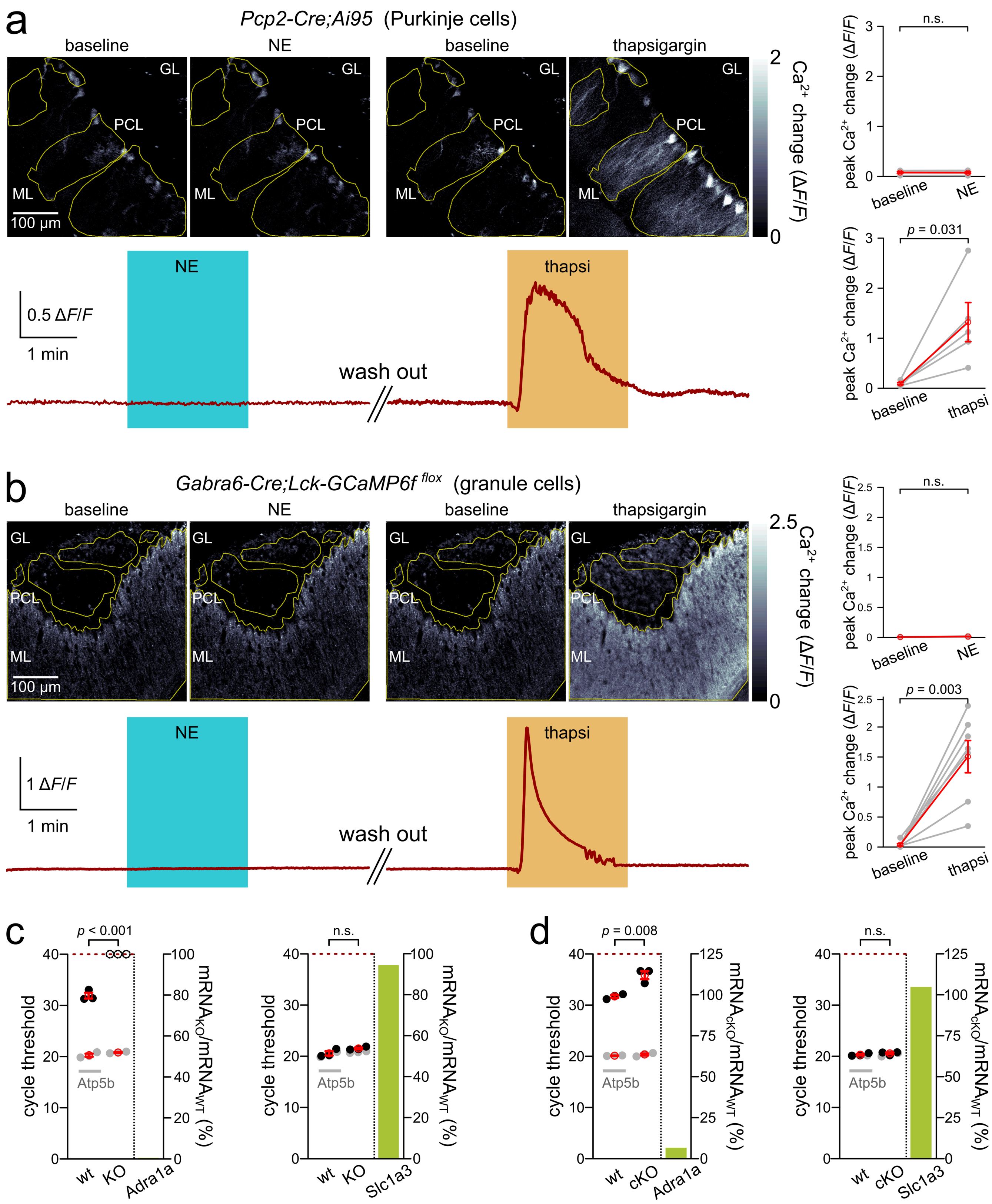

Supplementary Fig. 5 Astroglia-selective recombination in *Aldh1l1-CreER^{T2};Adra1a$^{cKO/cKO}$* mice leads to almost complete deletion of *Adra1a* mRNA in the cerebellum. a left, Responsiveness of Purkinje cells to bath application of NE (30 μM) and thapsigargin (TGN, 10 μM), respectively, in acute

cerebellar slices - 2P Ca^{2+} imaging of *Tg(Pcp2-cre)3555Jdhu/J;Ai95* mouse (postnatal day (PND) 30). Yellow circumferences indicate ROIs enclosing regions responsive to TGN. Trace represents mean Ca^{2+} change of all ROIs. Double-lines indicate blanking of 11 minutes of washout with aCSF. **right**, Comparison of mean Ca^{2+} change in response to NE ($t(4) = 1.425$) or TGN ($t(4) = -3.278$); red symbols indicate mean ± SEM, $n = 5$ slices; paired, two-tailed Student's *t*-test. **b left**, Same test as in a, but for granule cells in acute cerebellar slices - 2P Ca^{2+} imaging of *Tg(Gabra6-Cre)-B1Lfr;Lck-GCaMP6f^{flox}* mouse (PND 40). **right**, Comparison of mean Ca^{2+} change in response to NE ($t(6) = -2.131$) or TGN ($t(6) = -4.773$); red symbols indicate mean ± SEM, $n = 7$ slices; paired, two-tailed Student's *t*-test. **c**, Relative expression level of *Adra1a* (left) or *Slc1a3* (right) in *Adra1a$^{(+/+)}$* and *Adra1a$^{(-/-)}$* mice. Each plot displays cycle threshold (Ct) values on the left with average results from each mouse represented by a dot, red symbols indicate mean ± SEM, grey dots represent Ct values of the housekeeping gene Atp5b as reference and the red dotted line indicates the detection threshold. *Adra1a*: $t(4) = -14.145$. *Slc1a3:* $t(4) = -2.031$. $n = 3$ mice per condition; unpaired, two-tailed Student's *t*-test. Green bar, percent mRNA expression level in *Adra1a$^{(-/-)}$* compared to *Adra1a$^{(+/+)}$* mice. **d**, Same analysis as described in c, but for *Adra1a$^{(wt/wt)}$* compared to Adra1a$^{(cKO/cKO)}$ mice. *Adra1a*: $t(4) = -4.935$. *Slc1a3:* $t(4) = -1.072$. Red symbols indicate mean ± SEM, $n = 3$ mice per condition; unpaired, two-tailed Student's *t*-test. Green bar, percent mRNA expression level in *Adra1a$^{(cKO/cKO)}$* compared to Adra1a$^{(wt/wt)}$ mice. n.s., not significant. Source data are provided as a Source Data file.

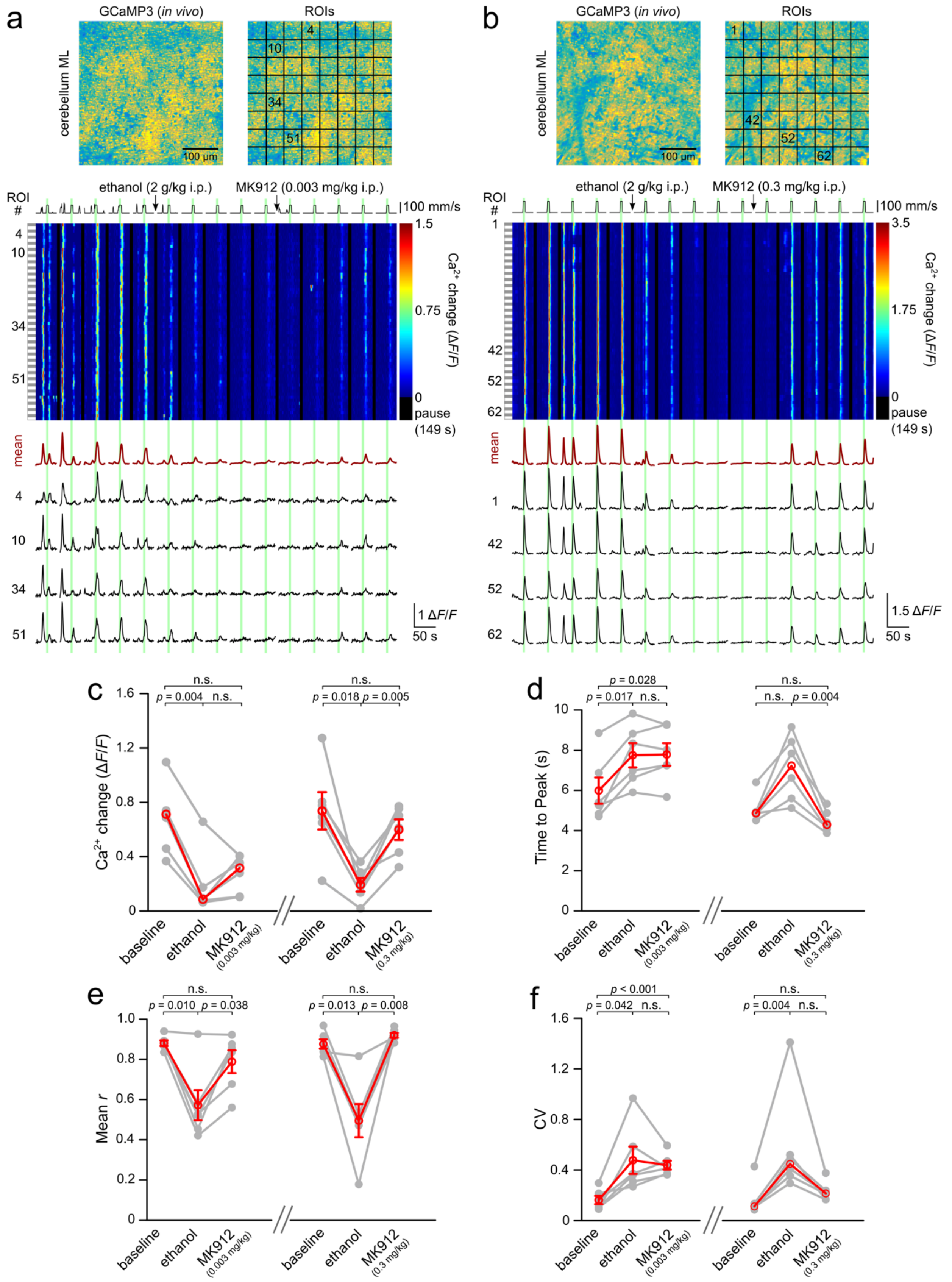

Supplementary Fig. 6 Pharmacological restoration of ethanol-inhibited vigilance-dependent cerebellar BG Ca^{2+} activation is dose-dependent. a upper, representative pseudocoloured *in vivo*

Ca^{2+} image of BG in tangential optical section of the cerebellum molecular layer and the respective ROIs for BG process clusters (6 independent experimental repetitions were obtained with similar results). **middle**, Pseudocoloured plots represent all ROIs' Ca^{2+} responses around consecutive enforced locomotion trials (green bars). Arrows highlight time points of i.p. injection of ethanol and MK912. **lower**, Corresponding average Ca^{2+} response of all ROIs (dark red) and Ca^{2+} dynamics traces from four representative BG process clusters highlighted above. **b** Same as in a; however, MK912 was i.p. injected at 0.3 mg/kg (6 independent experimental repetitions were obtained with similar results). **c - f** Population data representing effect of i.p. injection of ethanol (2 g/kg) followed by 0.003 mg/kg MK912 or 0.3 mg/kg MK912 on mean $\Delta F/F_{10s}$ (c), mean time from onset of locomotion to peak response (d), mean correlation coefficient (r) of Ca^{2+} responses among different ROIs (e) and mean coefficient of variation among different ROIs (f). Red symbols with error bars represent mean ± SEM and repeated measures ANOVA followed by Tukey-Kramer correction was applied. In the absence of error bars red symbols represent median and Friedman test followed by Tukey-Kramer correction was applied; n = 6 mice, respectively. Lines between dots connect values from same experiment. n.s., not significant. Source data are provided as a Source Data file.

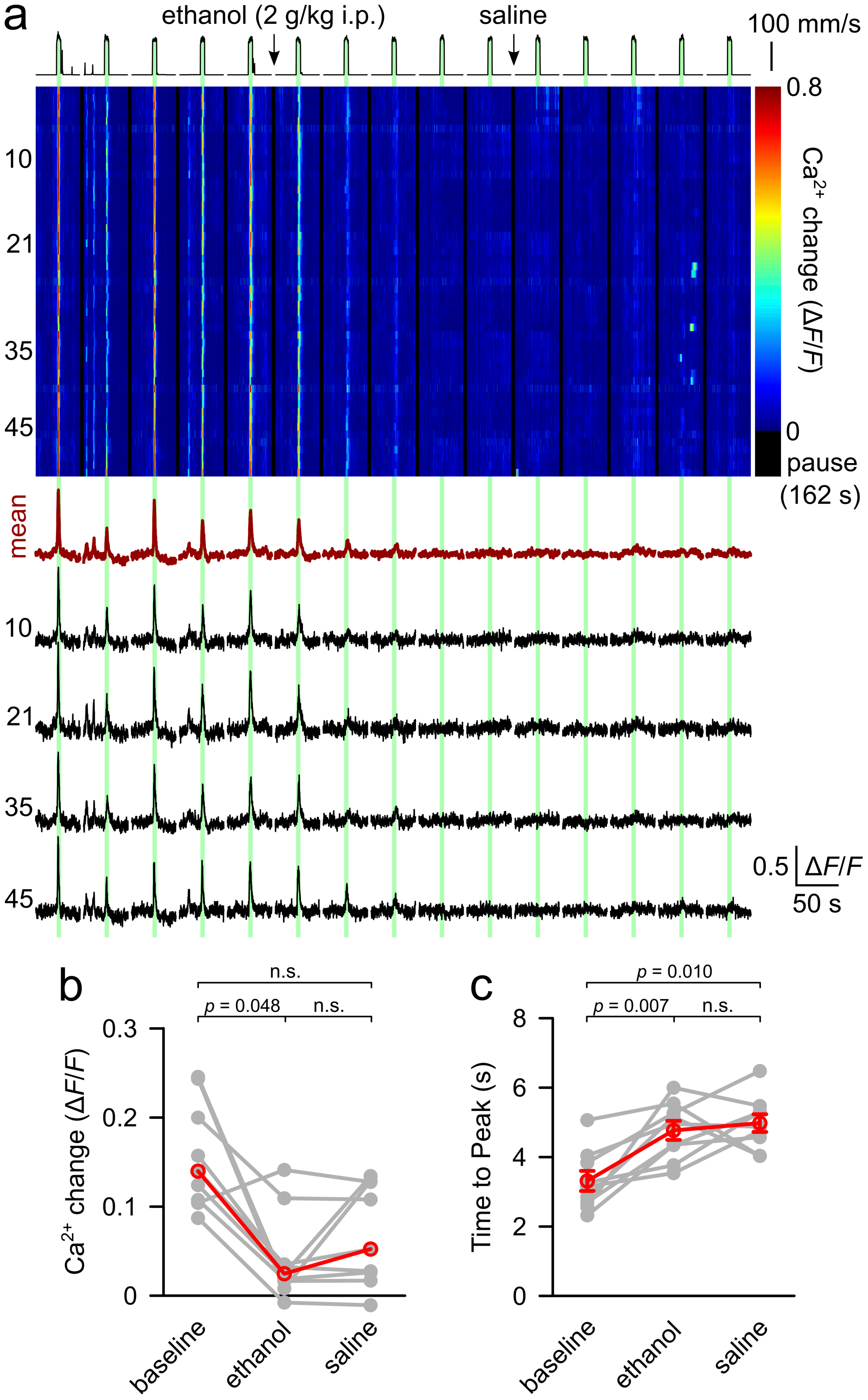

Supplementary Fig. 7 Persistence of inhibition of vigilance-dependent Ca^{2+} elevations in NE terminals by ethanol. a upper, Pseudocoloured plot represents Ca^{2+} responses of all ROI-defined

NE terminal clusters around consecutive enforced locomotion trials (green bars). Arrows highlight time points of i.p. injection of ethanol and saline. **lower**, Average Ca^{2+} response of all terminals (dark red) and traces of Ca^{2+} dynamics from four representative LC terminal clusters corresponding to previously highlighted pseudocoloured responses (upper). **b** Population data showing effects of ethanol (2 g/kg i.p.) and consecutive saline in the presence of ethanol on mean changes of locomotion-induced Ca^{2+} elevation. Red symbols represent median ($\Delta F/F$). n = 9 mice; Friedman test was followed by Tukey-Kramer correction. **c** Population data showing effects on time from onset of locomotion to peak amplitude. Red symbols represent mean ± SEM. n = 9 mice; repeated measures ANOVA ($F(2,16)$ = 11.655) was followed by Tukey-Kramer correction. n.s., not significant. Source data are provided as a Source Data file.

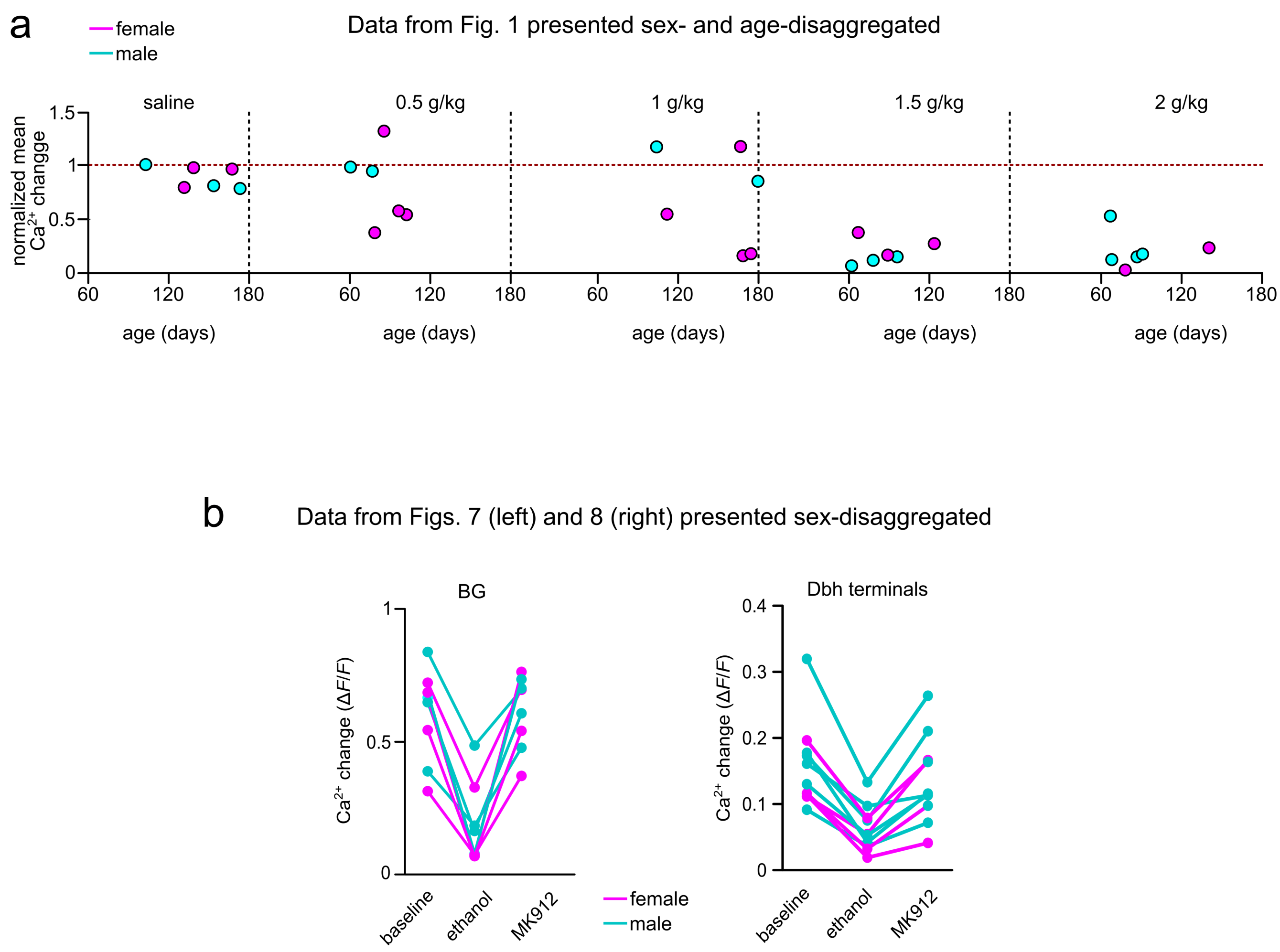

Supplementary Fig. 8 Assessment of ethanol sensitivity and effect of MK-912 disaggregated for sex and age. a Data from Fig. 1 presented spread out along the abscissa to represent the age of each mouse at the time the respective experiment was conducted and colourcoded to highlight the sex of the respective mouse. **b** Data from Figs. 7 (left) and 8 (right) presented colourcoded to highlight the sex of the mouse used for the respective experiment.

Primers	Sequences (5'-3')
For *Adra1a* in 2P imaging	
Adra1a-com-s	CGG GAT GGT TTT GGA TAA GA
Adra1a-wt-as	TCC CCA AAG CAG ACT ACT CG
Adra1a-mut-as	AGA CTG CCT TGG GAA AAG CG
For *Adra1a*^{cKO} in 2P imaging	
Adra1a-int2-s	AAC ATT TGG GGG ATG CAT AA
Adra1a-int2-as	CTG AGC TTT GCA GAG TGT GG
Adra1a-lox-s	GCG CAA CGC AAT TAA TGA TA
Adra1-int3-as	CGG AAT AAG CAG GGC ATT AG
For *Rosa26*-targeted (*R26-lsl-GCaMP3*, *Ai95* and *Lck-GCaMP6f*^{flox}) in 2P imaging	
Rosa26-5p-s	CTC TGC TGC CTC CTG GCT TCT
Rosa26-3p-as	CGA GGC GGA TCA CAA GCA ATA
CMV-E-as	TCA ATG GGC GGG GGT CGT T
For *Cre* in 2P imaging	
Cre-s	TGC CAC GAC CAA GTG ACA GCA ATG
Cre-as	ACC AGA GAC GGA AAT CCA TCG CTC
For RT-qPCR	
Adra1a-s	CCA TCT CCC TCG GTG AAA A
Adra1a-as	GCA CAG GTG GTT TCA TGG AT
Slc1a3-s	CCT GGG TTT TCA TTG GAG GGT TG
Slc1a3-as	GTG GCA GAA CTT GAG GAG GTC
Atp5b-s	TGA GAG AGG TCC TAT CAA AAC CA
Atp5b-as	CAC CAG AAT CTC CTG CTC AAC

Supplementary Table 1 List of primers used in this study
