# Supplementary material for: Ethanol abolishes vigilance-dependent astroglia network activation in mice by inhibiting norepinephrine release
Source: Nat Commun. 2020 Dec 2;11:6157. doi: 10.1038/s41467-020-19475-5 (PMC7710743; doi:10.1038/s41467-020-19475-5)
Supplement: Supplementary file 7 — Description of Additional Supplementary Files [file 41467_2020_19475_MOESM7_ESM.docx]

Description of Additional Supplementary Files

Title: supplementary movie 1

Description: Locomotion on Catwalk XT under baseline condition. Representative movie of Aldh1l1-CreERT2 mouse passing the Catwalk XT track in real time. Movie was taken from data presented and quantified in Fig. 10b and c.

Title: supplementary movie 2

Description: Locomotion on Catwalk XT 15 min following 2 g/kg i.p. ethanol exposure. Same mouse and experiment as shown in Supplementary Movie 1.

Title: supplementary movie 3

Description: Locomotion on Catwalk XT 45 min following 2 g/kg i.p. ethanol exposure. Same mouse and experiment as shown in Supplementary Movie 1.

Title: supplementary movie 4

Descriptions: Locomotion on Catwalk XT 90 min following 2 g/kg i.p. ethanol exposure. Same mouse and experiment as shown in Supplementary Movie 1.
