# Supplementary material for: Ethanol abolishes vigilance-dependent astroglia network activation in mice by inhibiting norepinephrine release
Source: Nat Commun. 2020 Dec 2;11:6157. doi: 10.1038/s41467-020-19475-5 (PMC7710743; doi:10.1038/s41467-020-19475-5)
Supplement: Supplementary file 8 — Reporting Summary [file 41467_2020_19475_MOESM8_ESM.pdf]

nature research

Corresponding author(s): Martin Paukert

Last updated by author(s): October 7, 2020

Reporting Summary

Nature Research wishes to improve the reproducibility of the work that we publish. This form provides structure for consistency and transparency in reporting. For further information on Nature Research policies, see our Editorial Policies and the Editorial Policy Checklist.

Please do not complete any field with "not applicable" or n/a. Refer to the help text for what text to use if an item is not relevant to your study. For final submission: please carefully check your responses for accuracy; you will not be able to make changes later.

Statistics

For all statistical analyses, confirm that the following items are present in the figure legend, table legend, main text, or Methods section.

n/a	Confirmed	
☐	☒	The exact sample size (*n*) for each experimental group/condition, given as a discrete number and unit of measurement
☐	☒	A statement on whether measurements were taken from distinct samples or whether the same sample was measured repeatedly
☐	☒	The statistical test(s) used AND whether they are one- or two-sided *Only common tests should be described solely by name; describe more complex techniques in the Methods section.*
☐	☒	A description of all covariates tested
☐	☒	A description of any assumptions or corrections, such as tests of normality and adjustment for multiple comparisons
☐	☒	A full description of the statistical parameters including central tendency (e.g. means) or other basic estimates (e.g. regression coefficient) AND variation (e.g. standard deviation) or associated estimates of uncertainty (e.g. confidence intervals)
☐	☒	For null hypothesis testing, the test statistic (e.g. *F*, *t*, *r*) with confidence intervals, effect sizes, degrees of freedom and *P* value noted *Give P values as exact values whenever suitable.*
☒	☐	For Bayesian analysis, information on the choice of priors and Markov chain Monte Carlo settings
☒	☐	For hierarchical and complex designs, identification of the appropriate level for tests and full reporting of outcomes
☐	☒	Estimates of effect sizes (e.g. Cohen's *d*, Pearson's *r*), indicating how they were calculated

Our web collection on statistics for biologists contains articles on many of the points above.

Software and code

Policy information about availability of computer code

Data collection	MATLAB R2011b or R2016a (Mathworks), ScanImage (v3.8.1 or v5.0; Vidrio Technologies, LLC), Labview2013 (version 13.0.1f2, National Instruments), Catwalk XT 8.1 software (Noldus)
Data analysis	MATLAB R2011b or R2016a (Mathworks), custom functions are provided as compressed folder and have been deposited here: https://github.com/PaukertLab/NCOMMS-20-01106/find/main

For manuscripts utilizing custom algorithms or software that are central to the research but not yet described in published literature, software must be made available to editors and reviewers. We strongly encourage code deposition in a community repository (e.g. GitHub). See the Nature Research guidelines for submitting code & software for further information.

Data

Policy information about availability of data

All manuscripts must include a data availability statement. This statement should provide the following information, where applicable:

- Accession codes, unique identifiers, or web links for publicly available datasets
- A list of figures that have associated raw data
- A description of any restrictions on data availability

The datasets generated and/or analyzed during the current study are provided as source data file (https://datadryad.org/stash/dataset/doi:10.5061/dryad.08kprr516).

Field-specific reporting

Please select the one below that is the best fit for your research. If you are not sure, read the appropriate sections before making your selection.

☒ Life sciences ☐ Behavioural & social sciences ☐ Ecological, evolutionary & environmental sciences

For a reference copy of the document with all sections, see nature.com/documents/nr-reporting-summary-flat.pdf

Life sciences study design

All studies must disclose on these points even when the disclosure is negative.

Sample size	Sample sizes were determined based on comparable studies published previously, including ours. (PMID 24945771, 28742117)
Data exclusions	In in vivo experiments (Figs. 1, 2, 3, 4b, 6, 7, 8, 9 and Supplementary Figs. 1, 2, 4a, 5a,b, 6, 7) a pre-established custom-written MATLAB script was used to objectively identify enforced locomotion trials that were preceded by voluntary locomotion events (criterion: three consecutive image frames exceeded 3x baseline standard deviation). Such "contaminated" trials were excluded from analysis.
Replication	For functional experiments minimum n = 3 (Supplementary Fig. 1); usually at least n = 6 yielding successfully similar results. For descriptive immunocytochemistry all staining results were obtained three times with similar results. All replication numbers have been added to the respective figure legend.
Randomization	The sex of the mice was distributed randomly based on availability of transgenic mice.
Blinding	The enforced locomotion behavioral paradigm is highly automated. In addition, the analysis is run using predefined MATLAB scripts with identical scripts being applied to all experimental conditions. Therefore, due to the experimental design including data analysis, any opportunity for investigator bias was minimized.

Reporting for specific materials, systems and methods

We require information from authors about some types of materials, experimental systems and methods used in many studies. Here, indicate whether each material, system or method listed is relevant to your study. If you are not sure if a list item applies to your research, read the appropriate section before selecting a response.

Materials & experimental systems

n/a	Involved in the study	
☐	☒	Antibodies
☒	☐	Eukaryotic cell lines
☒	☐	Palaeontology and archaeology
☐	☒	Animals and other organisms
☒	☐	Human research participants
☒	☐	Clinical data
☒	☐	Dual use research of concern

Methods

n/a	Involved in the study	
☒	☐	ChIP-seq
☒	☐	Flow cytometry
☒	☐	MRI-based neuroimaging

Antibodies

Antibodies used	The primary antibodies were used at following concentrations: chicken anti-eGFP (1:1000, # A10262, polyclonal, Thermo Fisher Scientific) (Figs. 4a and 8a) and mouse (IgG1) anti-S100β (1:500, # MA1-25005, monoclonal (SH-B4), Thermo Fisher Scientific) (Fig. 4a) or rabbit anti-DBH (1:500, # 22806, polyclonal, ImmunoStar) (Fig. 8a). Secondary antibodies (all used at 1:5000 dilution and purchased from Jackson ImmunoResearch): Alexa Fluor® 488-conjugated AffiniPure goat anti-Chicken IgY (#103-545-155), Alexa Fluor® 647-conjugated AffiniPure Goat Anti-Mouse IgG1 (#115-605-205), Alexa Fluor® 647 AffiniPure Goat Anti-Rabbit IgG (H+L) (#111-605-144).
Validation	Validation of primary antibodies: (1) anti-eGFP: when this antibody was used at the same dilution and staining conditions on tissue that did not express GCaMP6f, the entire immunostaining looked like the granuar layer (GL) stained for GCaMP6f in Fig. 4a left bottom. Continued in Reporting Summary Extension file.

Animals and other organisms

Policy information about studies involving animals; ARRIVE guidelines recommended for reporting animal research

Laboratory animals	This information is contained in the Reporting Summary Extension file.
Wild animals	No wild animals were used.
Field-collected samples	No field collected samples were used.
Ethics oversight	All animal procedures were conducted in accordance with guidelines and protocols of the University of Texas Health Science Center at San Antonio (UTHSCSA) Institutional Animal Care and Use Committee.

Note that full information on the approval of the study protocol must also be provided in the manuscript.

Dual use research of concern

Policy information about dual use research of concern

Hazards

Could the accidental, deliberate or reckless misuse of agents or technologies generated in the work, or the application of information presented in the manuscript, pose a threat to:

No	Yes	
☒	☐	Public health
☒	☐	National security
☒	☐	Crops and/or
☒	☐	livestock Ecosystems
☒	☐	Any other significant area

Experiments of concern

Does the work involve any of these experiments of concern:

No	Yes	
☒	☐	Demonstrate how to render a vaccine ineffective
☒	☐	Confer resistance to therapeutically useful antibiotics or antiviral
☒	☐	agents Enhance the virulence of a pathogen or render a nonpathogen
☒	☐	virulent Increase transmissibility of a pathogen
☒	☐	Alter the host range of a pathogen
☒	☐	Enable evasion of diagnostic/detection modalities
☒	☐	Enable the weaponization of a biological agent or
☒	☐	toxin
		Any other potentially harmful combination of experiments and agents

[illegible]is checklist template is licensed under a Creative Commons Attribution 4.0 International License, which permits use, sharing, adaptation, distribution and reproduction in any medium or format, as long as you give [illegible]ppropriate credit to the original author(s) and the source, provide a link to the Creative Commons license, and indicate if changes were made. The images or other third party material in this article are included in th[illegible] [illegible]ticle's Creative Commons license, unless indicated otherwise in a credit line to the material. If material is not included in the article's Creative Commons license and your intended use is not permitted by statutory [illegible]
